# Supplementary material for: Dihydroquercetin Supplementation Improved Hepatic Lipid Dysmetabolism Mediated by Gut Microbiota in High-Fat Diet (HFD)-Fed Mice
Source: Nutrients. 2022 Dec 7;14(24):5214. doi: 10.3390/nu14245214 (PMC9788101; doi:10.3390/nu14245214)
Supplement: Supplementary file 1 [file nutrients-14-05214-s001.zip › nutrients-2043974-supplementary/Supplementary material.pdf]

Supplementary Figures

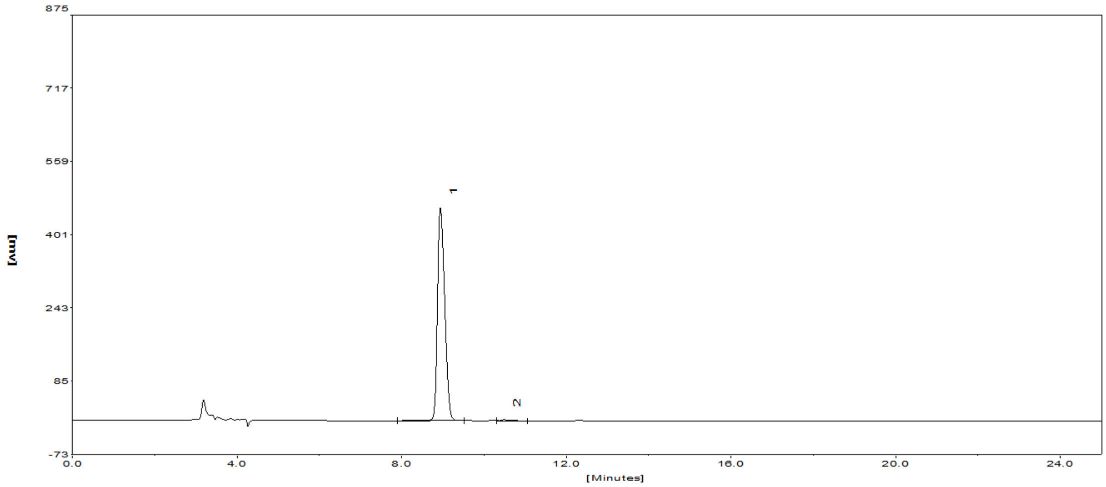

Figure S1. Total ion chromatography (TIC) of DHQ.

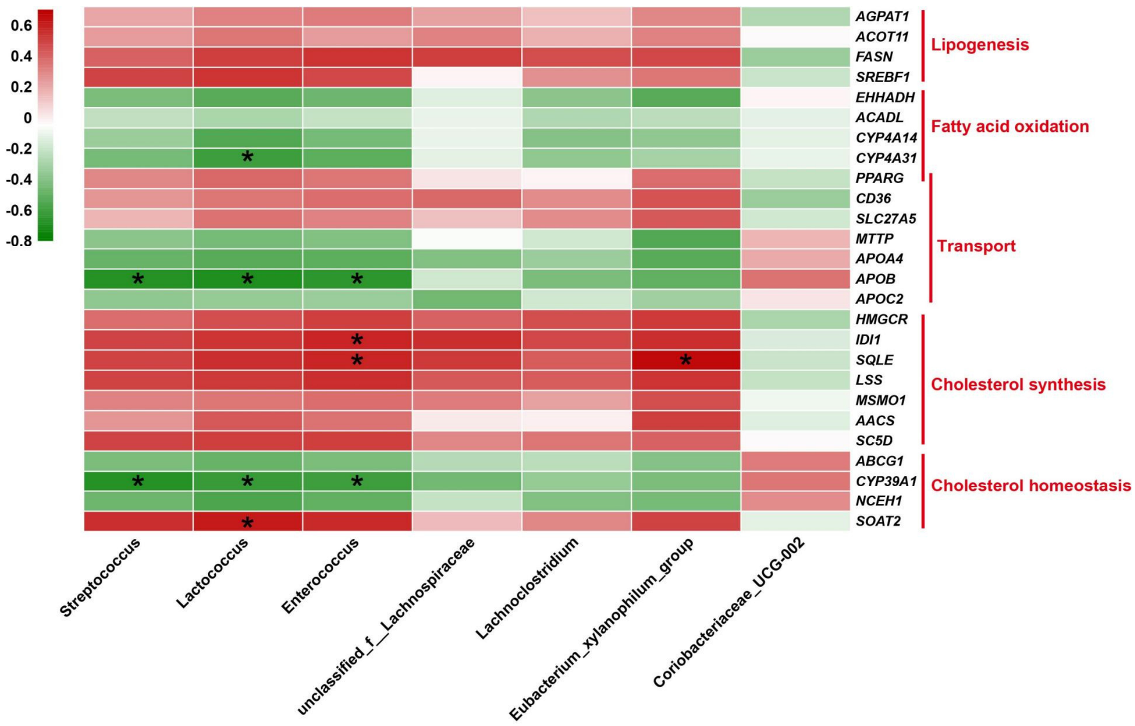

Figure S2. Pearson's correlation analysis between fecal microbiota at the genus level and hepatic lipid metabolic related genes.
